# Malignant pilomatricoma with multiple bone metastases in a dog: Histological and immunohistochemical study

**DOI:** 10.3892/etm.2013.974

**Published:** 2013-02-25

**Authors:** MANUELA MARTANO, LUIGI NAVAS, LEONARDO MEOMARTINO, FRANCESCA ABRAMO, BRUNELLA RESTUCCI, PAOLA MAIOLINO, LORENZO LO MUZIO

**Affiliations:** 1 Department of Pathology and Animal Health, Faculty of Biotechnological Sciences, University Federico II -Via Delpino, Naples I-80137;; 2 Department of Veterinary Clinical Science, University Federico II -Via Delpino, Naples I-80137;; 3 Department of Animal Pathology, University of Pisa, Viale delle Piagge 2, Pisa I-56124;; 4 Department of Pathology and Animal Health, Faculty of Veterinary Medicine, University Federico II -Via Delpino, Naples I-80137;; 5 Department of Clinical and Experimental Medicine, Faculty of Medicine, University of Foggia, Foggia I-71121, Italy

**Keywords:** malignant pilomatricoma, dog, immunohistochemistry

## Abstract

An eleven year-old mongrel dog was referred with a history of left forelimb lameness and an ulcerated mass on the neck. Histologically, the cutaneous neoplasm revealed cystic lobules composed of basaloid cells with abrupt transition to central keratotic material, containing pycnotic and shadow cells. Approximately 3 months after primary diagnosis, a lesion of the cortical bone on the left humerus was observed using X-ray. Samples obtained from the humerus were processed for histopathological examination and the neoplastic tissue was observed to be similar to the type identified in the neck. Based on these findings, the tumor was diagnosed as a malignant pilomatricoma (MP) with bone metastasis. MP is a rare skin tumor that originates from hair matrix cells. To date, only nine reports have been presented in dogs. In the present study, we discuss the cytological and histological patterns of MP, confirmed by immunohistochemistry using β catenin antibody.

## Introduction

Pilomatricoma is a benign adnexal neoplasm with follicular differentiation similar to matrical cells of the hair bulb, which occurs in middle-aged to older dogs (2–7 years of age) with normal gender distribution (1–5). Pilomatricoma manifests as an asymptomatic dermal or subcutaneous mass with alopecia of overlying skin, usually situated on the scalp, posterior area of the neck, buttocks and on the upper extremities (6,7). Breeds with continuous hair growth (Kerry Blue Terriers, Poodles, Bedlington Terriers, Bichon Frisés and Schnauzers) have higher susceptibility due to the greater mitotic activity of their hair follicles (7). Although benign pilomatricoma is a relatively frequent adnexal tumor (3% of all epithelial skin tumors) the incidence of its malignant counterpart [matrical carcinoma or malignant pilomatricoma (MP)] is extremely low (7–9). To the best of our knowledge, only nine reports of MP have been reported thus far in dogs (10–17), most of which presented metastasis in bone (10,12–16). The localization and the clinical features of MP are similar to that of its benign counterpart, with the exception of its tendency to recur following incomplete excision and to metastasize to the underlying bone through contiguity or to distant sites (7).

## Case report

**Figure 1:**
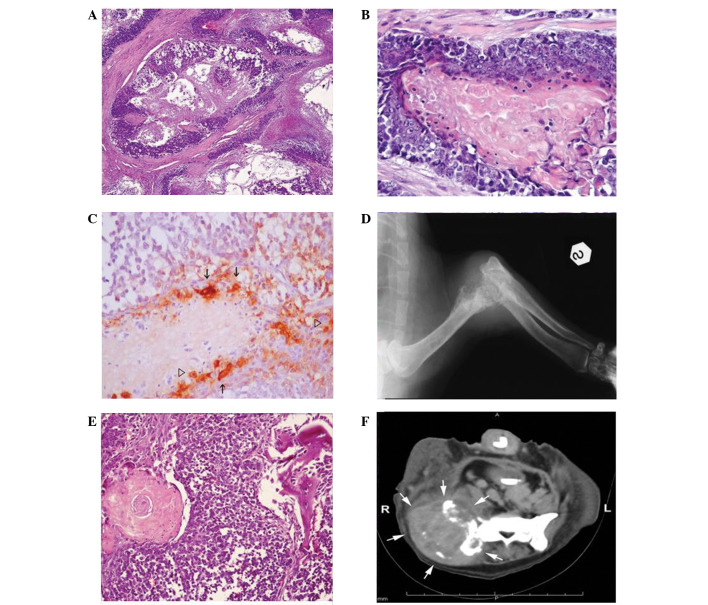
Microscopic, X-ray and CT images of malignant pilomatricoma with bone metastases. (A) Skin from the neck of the dog. Microscopic view of the primary skin lesion: irregular lobules composed of proliferating basaloid cells organized around ghost cells (hematoxylin and eosin staining). (B) Higher magnification of (A); basaloid cells with pleomorphism, vesicular nuclei and prominent nucleoli (hematoxylin and eosin staining). (C) Skin from the neck of the dog. Irregularly distributed β catenin immunoreactivity in the cytoplasm of basaloid cells along the central cyst (arrows) and scattered positive nuclei (arrowheads; immunohistochemical method: streptavidin-biotin peroxidase). (D) Lateral X-ray view of left elbow. Marked permeative osteolysis with severe soft tissue enlargement on the distal humerus. (E) Metastatic lesion of the humerus. A high proportion of atypical basaloid cells associated with a ghost cell aggregate (left side) and bone lysis (right side; hematoxylin and eosin staining). (F) Transverse post-contrast CT scan, taken at level of the iliac crests. On the right crest, there is disomogeneous soft tissue mass, with mineralized spots, periosteal interrupted proliferations and moth-eaten osteolysis (arrows).

An eleven year-old male mongrel dog was referred with a history of a left forelimb lameness, treated with non-steroidal anti-inflammatory drugs, without improvement. On physical examination, a painful zone near the left elbow was observed, without any abnormality demonstrated by radiography. An ulcerated mass on the neck was also detected. The mass was 4 cm in diameter, well circumscribed, firm and not painful on palpation. Cytological examination of samples obtained by fine needle aspiration showed small cohesive epithelial aggregates composed of basaloid cells, a small amount of amorphous keratinized material and a few ghost cells. Due to these findings, an initial diagnosis of a follicular tumor with matrical differentiation was made. The mass was excised under general anesthesia, fixed in 10% formalin and routinely processed for histopathological examination. Microscopic examination revealed an epithelial neoplasia localized in the dermis and subcutaneous tissue, without connection to the overlying epidermis. The tumor was characterized by irregular cystic lobules, within which neoplastic cells were arranged in a circular configuration, with nucleated basaloid-type cells on the periphery and pink amorphous keratin with enucleated shadow cells in the centre (Fig. 1A). There was an abrupt transition between the basaloid cells and the central core. The basaloid cells had large, vesicular, hyperchromatic nuclei with prominent nucleoli and a moderate amount of clear cytoplasm and the centrally located cells showed well-delineated cell borders and a central unstained area corresponding to the lost nucleus (Fig. 1B). Scattered throughout the basaloid cell layer, 3–4 mitotic figures were observed at high power field (×400 magnification), some of which were atypical. In certain areas inflammatory cells, including mainly lymphocytes, plasma cells, macrophages and foreign body giant cells, were observed. The histological features were consistent with an MP.

To better define the matrical differentiation of this tumor, immunohistochemistry was performed by the streptavidin-biotin peroxidase complex method using a commercially available antibody against β catenin (monoclonal mouse anti-human, clone E-5, sc-7963; Santa Cruz Biotechnology, Heidelberg, Germany). The reaction of β catenin in the neoplastic tissue was observed in the cytoplasm of certain centrally located well-differentiated keratinocytes and in the nuclei of some of the basaloid cells. In the overlying epidermis that served as a control, β catenin was shown to be expressed in the keratinocytes in a perimembranous pattern (Fig. 1C).

After one month the dog presented with a severe lameness in the same left limb. Radiography of the elbow showed a severe permeative osteolysis with a long transitional zone of the distal epiphysis of the left humerus (Fig. 1D). A bone biopsy was performed and a neoplastic lesion similar to those described for the primary cutaneous tumor was observed. Due to these staging results, amputation was performed. A nodular bone mass was observed on the excised bone. The entire bone mass, which was 4 cm in diameter, white and friable, was fixed in 10% formalin for histopathology. Upon microscopic examination, performed following the routine procedure for decalcification, a neoplasia similar to the type identified in the primary skin mass was observed. It was confined to the bone with associated areas of bone lysis, mineralization, chondroid and osseous metaplasia (Fig. 1E). Compared with the primary tumor, there were larger areas of necrosis and a greater proportion of basaloid cells, which showed more severe atypical features. Immunohistochemical findings using the β catenin antibody showed the presence of scattered cells with cytoplasmic positivity and nuclear staining in the majority of the basaloid cells. All the results derived from clinical, surgical, histological and immunohistochemical investigations strongly supported the diagnosis of a metastatic MP.

The first five-month follow-up was excellent but, one month later, the dog presented again due to hind limb lameness and skin lesions. On clinical evaluation, multiple palpable masses were appreciable on the skin of the right forehead, the skin of the left mandible, right humerus, left ribs (from third to seventh), right ileus, right femur, left knee and both tarsi. All the lesions were CT scanned and were characterized by a dishomogeneous pattern with mineralized spots, periosteal interrupted proliferations and moth-eaten osteolysis (Fig. 1F). No lung or abdominal metastasis was appreciable by thoracic radiography and abdominal ultrasonography, respectively.

Due to the extremely poor prognosis, euthanasia but not necropsy was permitted by the owner.

## Discussion

The histopathological features of pilomatricoma are characterized by irregularly shaped, lobulated islands of a dual cell population of basaloid cells and shadow or ghost cells, which represent keratinized immature hair cells. Within the lobules, an abrupt keratinization from basaloid to shadow cells is characteristic of this neoplasia (3,5).

Histologically, the criteria for malignancy in the differential diagnosis between MP and its benign counterpart are the presence of an encapsulated asymmetric ulcerated tumor growth, increased mitotic figures with nuclear atypical features, infiltration of the adjacent skin and lymphatic invasion at the periphery of the mass (3,7,8).

Since almost all the above histological findings were observed in the present case, the diagnosis of MP was made. The malignant behavior of the tumor was confirmed subsequently by the detection of multiple bone metastases. In addition, immunohistochemical reactivity to β catenin confirmed the diagnosis of MP.

β catenin is a 92-kDa-sized cytoplasmic protein, involved in intercellular adhesion and the Wnt-signaling pathway. The cellular localization of β catenin is determined by its phosphorylation state. At the cell surface, as a subunit of the cadherin complex, it interacts with E cadherin, linking it to the actin cytoskeleton to create the cell-cell adherens junctions. When this protein is free in the cytosol, it is constitutively phosphorylated and directed to the nucleus for destruction (18). Deregulation of the Wnt/β catenin pathway, attributable to abnormalities of CTNNB1, the gene encoding β catenin, has been recognized to prevent phosphorylation of β catenin, resulting in its accumulation in the nucleus. At the nucleus, β catenin interacts with Tcf/Lef transcription factors to provide a stimulus for cell proliferation and differentiation, and most likely for neoplastic transformation (19). Numerous human studies have demonstrated that mutation of CTNNB1 is a frequent cause of Wnt signaling pathway activation in pilomatricoma (20,21).

In skin tumors the variability of membrane, cytoplasmic and nuclear staining of β catenin is great, however, in pilomatricoma and its malignant counterpart an intense and diffuse nuclear pattern in the proliferating matrix (basaloid) cells is the main finding in the majority of the literature (22).

In the present case we observed a predominant nuclear staining of β catenin in basaloid cells, while a few transitional cells showed a prevalent membrane-associated reactivity.

In conclusion, this study documents a case of canine MP and raises awareness of the potential aggressiveness of matrical tumors. In addition, the immunohistochemical observations suggest that β catenin is involved in the pathogenesis of this canine neoplasm and may be a useful diagnostic marker for MP in dogs, as previously demonstrated in humans.
